# Magnetic Micro-Solid-Phase Extraction Using a Novel Carbon-Based Composite Coupled with HPLC–MS/MS for Steroid Multiclass Determination in Human Plasma

**DOI:** 10.3390/molecules26072061

**Published:** 2021-04-03

**Authors:** Andrea Speltini, Francesca Merlo, Federica Maraschi, Giorgio Marrubini, Anna Faravelli, Antonella Profumo

**Affiliations:** 1 Department of Drug Sciences, University of Pavia, 27100 Pavia, Italy; giorgio.marrubini@unipv.it; 2 Department of Chemistry, University of Pavia, 27100 Pavia, Italy; francesca.merlo02@universitadipavia.it (F.M.); federica.maraschi@unipv.it (F.M.); anna.faravelli01@universitadipavia.it (A.F.)

**Keywords:** bioanalysis, carbon materials, HPLC–MS, biological matrices, solid-phase extraction, sample preparation

## Abstract

A micron-sized sorbent, Magn-Humic, has been prepared by humic acids pyrolysis onto silica-coated magnetite. The material was characterized by scanning electron microscopy (SEM), transmission electron microscopy (TEM), energy dispersive spectroscopy (EDS), thermogravimetric analysis (TGA), and Brunauer, Emmett, and Teller (BET) surface area measurements and applied for simultaneous magnetic solid-phase extraction (MSPE) of glucocorticoids, estrogens, progestogens, and androgens at ng mL^−1^ levels from human plasma followed by high-performance liquid chromatography coupled with mass spectrometry (HPLC–MS/MS). Due to the low affinity for proteins, steroids extraction was done with no need for proteins precipitation/centrifugation. As highlighted by a design of experiments, MSPE was performed on 250 µL plasma (after 1:4 dilution) by 50 mg Magn-Humic (reusable for eight extractions) achieving quantitative recovery and satisfying clean-up. This was improved by washing (2 mL 2% *v*/*v* formic acid) prior to analytes elution by 0.5 mL 1:1 *v*/*v* methanol-acetonitrile followed by 0.5 mL methanol; eluate reduction to 0.25 mL compensated the initial sample dilution. The accuracy was assessed in certified blank fetal bovine serum and in human plasma, gaining satisfactory recovery in the range 65–122%, detection limits in the range 0.02–0.3 ng mL^−1^ (0.8 ng mL^−1^ for 17-β-estradiol) and suitable inter-day precision (relative standard deviation (RSD) <14%, *n* = 3). The method was evaluated in terms of selectivity, sensitivity, matrix-effect, instrumental carry-over, and it was applied to human plasma samples.

## 1. Introduction

The search for new sample preparation procedures with improved throughput, simple workflow, and reduced use of organic solvents is nowadays one of the most desired goals in analytical sample treatment. This is especially important in the case of complex matrices such as environmental, food, and biological samples, wherein in most cases the target analytes are present at very low concentrations together with huge amounts of other matrix constituents [1,2,3,4]. These are major interfering species in various steps of the analytical protocol, from analyte isolation to final instrumental quantitation, thus working procedures for extraction, clean-up and, possibly, pre-concentration, are increasingly required.

With regard to sample treatment for biological matrices, proteins (up to 80 g L^−1^) are the major interferents in plasmatic steroids determination, calling for pretreatments such as sample dilution and proteins precipitation [5,6,7,8,9,10,11,12,13] before extraction that is done, for instance, by solid-phase extraction (SPE) [5,7,8,9,12,13]. Quantitation is today mainly performed by high-performance liquid chromatography coupled with mass spectrometry detection (HPLC–MS), which ensures selective and sensitive determination [7,8,9,12,14,15,16]; some methods also involving gas chromatography coupled with MS detection have been proposed, necessarily requiring analytes derivatization before analysis [13,17].

In this context, our previous works showed the versatility of the mixed-mode HA-C@silica sorbent, employed in conventional SPE cartridges for enrichment/clean-up of various compounds in environmental and biological matrices prior to HPLC–MS [18,19,20,21]. With regard to the biomatrix, HA-C@silica proved to be advantageous due to its protein exclusion [21].

Besides conventional column SPE, magnetic solid-phase extraction (MSPE) has emerged in the last decades as a promising and a straightforward sample preparation technique due to simple and quick extraction [1,2]. In dispersive MSPE, the magnetic sorbent is dispersed under agitation in the sample solution, providing high surface contact and full interaction between the analytes and the sorbent particles, and then it is easily isolated from the solution using a small magnet [2].

Based on these advantages, MSPE has been adopted in the last years as sample treatment for determination of various pharmaceuticals in biological matrices, mostly antibiotics, antidepressants, narcotic analgesics, benzodiazepines, and anti-inflammatory drugs [2]. However, it should be noted that only very few MSPE-based methods, entailing use of gold-modified nanoparticles, have been reported to extract some selected steroids from human plasma [6] and urine [6,22]. Based on the above discussion and in light of our earlier research [21], in this study a novel magnetic sorbent (Magn-Humic) has been prepared by pyrolysis of humic acids (HAs) onto silica-coated magnetite. The siliceous shell, grown up on magnetite (Fe_3_O_4_) by a sol-gel procedure, was conveniently exploited to support HAs before pyrolysis, yielding a micron-sized magnetic sorbent relying on the sorption properties of the HA-derived carbon phase, to be easily used in human plasma for batch-extraction of steroids, namely prednisolone (PREDLO), prednisone (PRED), hydro-cortisone (H-CORT), cortisone (CORT), betamethasone (BETA), dexamethasone (DEXA), triamcinolone (TRIAM), 17-β-estradiol (E2), testosterone (TST), epitestosterone (EPI), 17-α-ethynylestradiol (EE2), estrone (E1), hydroxyprogesterone (H-PROG), fluocinolone (FLUO), progesterone (PROG), and medroxyprogesterone acetate (M-PROG).

The material was characterized by various techniques, namely thermogravimetric analysis (TGA), scanning electron microscopy (SEM), transmission electron microscopy (TEM), energy dispersive spectroscopy (EDS), and surface area measurements by Brunauer, Emmett, and Teller (BET) method. Preliminary protein exclusion tests and extractions were done in bovine serum albumin (BSA) solution, and then the MSPE was moved in a real biological sample, i.e., certified hormone-free fetal bovine serum (FBS). A simple MSPE was developed and optimized by a design of experiments (DoE) to extract steroids while achieving sample clean-up and preconcentration prior to HPLC–MS/MS. The proposed method, assessed by the main figures of merit and compared with the currently available procedures based on SPE before instrumental analysis, was tested in human plasma and applied to multiclass steroids determination in blind plasma samples.

## 2. Results and Discussion

### 2.1. Magn-Humic Characterizations

**Figure 1:**
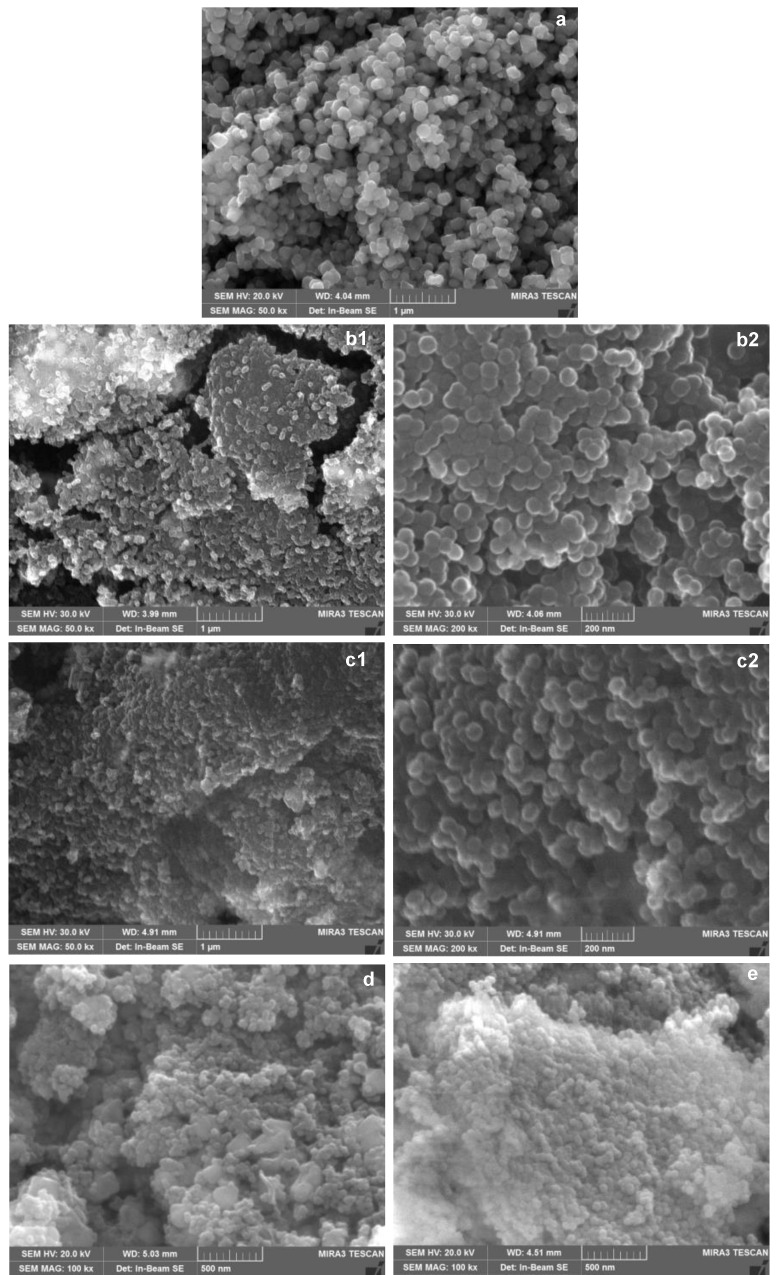
Representative scanning electron microscopy (SEM) images acquired on (**a**) pristine Fe_3_O_4_, (**b1**,**b2**) SiO_2_@Fe_3_O_4_, (**c1**,**c2**) c-SiO_2_@Fe_3_O_4_, (**d**) Magn-Humic, and (**e**) c-Magn-Humic.

The morphology of the prepared materials was appraised by SEM. As shown in Figure 1, the Fe_3_O_4_ nanoparticles characterized by definite edges (Figure 1a) have been coated after sol-gel by sphere-like silica particles, with a better homogeneity in shape and dimension observed on SiO_2_@Fe_3_O_4_ (Figure 1(b1,b2)) compared to that air-calcined after sol-gel, c-SiO_2_@Fe_3_O_4_ (Figure 1(c1,c2)).

The siliceous coating on the magnetic core was better evidenced by TEM (average thickness 10–20 nm) and confirmed by the Si/O and Si/Fe ratios from compositional EDS analysis, which showed a homogeneous distribution of the elements (see Supplementary Information). The SEM images of Magn-Humic (Figure 1d) and c-Magn-Humic (Figure 1e) evidenced a more compact structure of the latter—in agreement with the lower surface area, showed hereafter—and some carbon structures more visible in Magn-Humic. The overall procedure yielded micrometric materials, with particles ranging from few to some tens of microns, as shown by the additional SEM images in Supplementary Material.

The amount of carbon phase in the composites, determined by TGA, resulted to be 2.0 and 3.3 wt% for c-Magn-Humic and Magn-Humic, respectively. To achieve accurate quantitation of the carbonaceous fraction, the weight losses of c-SiO_2_@Fe_3_O_4_ and SiO_2_@Fe_3_O_4_—used as “blank” samples—were subtracted to those of the respective sorbents. In the case of SiO_2_@Fe_3_O_4_, prepared with no calcination, an isothermal pretreatment of the sample (320 °C, 12 h) was necessary to remove cetyltrimethylammonium bromide (CTAB) entrapped in the silica shell. This was necessary because the great weight loss due to the surfactant release during the sample heating overlapped the weight loss between 320 °C and 600 °C of Magn-Humic related to the pyrolyzed HAs, making the calculation of the actual carbon phase wt% impracticable. TGA profiles are shown in Supplementary Information.

Surface area data, mean values from three measurements on each sample, are shown in Table 1.

As apparent, surface area was enlarged compared to pristine magnetite (20–50 m^2^ g^−1^) due to the formation of the silica shell by sol-gel, and this increase is more evident performing calcination (see c-SiO_2_@Fe_3_O_4_), which removes CTAB [23,24]. Instead, the deposition of pyrolyzed HAs on c-SiO_2_@Fe_3_O_4_ induces a decrease of surface area because of carbon structures growing in the silica pores. This turns into agreement with the preparation of HA-C@silica [18,19]. For SiO_2_@Fe_3_O_4_, after pyrolysis surface area showed a remarkable increase justifiable considering that CTAB is anyhow released during the pyrolytic treatment (600 °C). These findings fit with the TGA results above discussed and underline that the preparation of Magn-Humic is doubly advantageous as calcination after sol-gel can be avoided obtaining, in any case, a carbon-based magnetic material with higher surface area.

### 2.2. Protein Exclusion and Explorative Extraction Tests

In the first part of this study, the prepared materials were investigated for their affinity toward proteins, according to the studies of restricted access carbon nanotubes (RACNTs) for clean-up of biological matrices [25,26,27]. Protein exclusion tests were here performed in batch (rotating plate, 170 rpm, 3 min) by contacting 50 mg sorbent with 1 mL PBS containing 7 mg BSA [21], a quantity below saturation [26,27] and however lower compared to those of biological samples (see Section 2.4). The excluded protein—not retained on the solid phase—was quantified by UV-Vis spectrophotometry (spectra acquisition 200–800 nm, quantification at λ_max_ 280 nm) [21,26], and results are shown in Table 2.

As apparent, up to 90–95% of the sample BSA is excluded by the two magnetic materials, in good agreement with the behavior of HA-C@silica and with a performance similar to that experimentally observed on RACNTs [21]. As discussed more in-depth in previous work [21], also in line with Mullet and Pawlyszin [28], the low affinity of the sorbents for proteins is essentially due to the small amount of carbon phase (2–3%, by TGA) joined to the low hydrophobicity imparted by oxygenated groups embedded in the carbon phase [18,19] that hamper protein retention. Predictably, protein exclusion was almost quantitative (97%) on Fe_3_O_4_ as control sample.

Both magnetic composites were tested for explorative MSPE experiments (in duplicate) by contacting 50 mg of sorbent with 2 mL BSA solution (10 g L^−1^, 0.01 M phosphate buffer solution, PBS, pH 7.2 [21]), spiked with 2 mg L^−1^ of CORT, E2, TST, and PROG as probes. After extraction (3 min vortex, 1400 rpm), the sorbent was washed with 2 mL 2% formic acid (FA) followed by 2 mL 30% methanol (MeOH) aqueous solutions, and analytes were eluted in vortex by 2 × 1 mL MeOH-acetonitrile (ACN) (1:1) [21] and quantified by HPLC–UV (see Appendix A). Higher recoveries (in the range 40–76%) were observed for Magn-Humic compared to c-Magn-Humic (between 18 and 52%). At the same time, control tests on the intermediate materials (recovery 16–56% and 1–13% for SiO_2_@Fe_3_O_4_ and c-SiO_2_@Fe_3_O_4_, respectively) proved the major role of the carbonaceous phase deriving from HAs pyrolysis, able to retain steroids by a mixed-mode mechanism relying on π stacking and polar–apolar balanced interactions [18,20]. As expected, pristine magnetite did not show retention capability for the steroids, which were not quantifiable in the MSPE eluate (< 0.2 mg L^−1^).

In light of these explorative recovery tests, protein exclusion data and results from physical-chemical characterization, Magn-Humic prepared with no calcination after sol-gel was selected for in-depth investigation.

### 2.3. Development of the MSPE Procedure in BSA Solution Using Magn-Humic

For the MSPE development, experiments were undertaken in solution of BSA (10 g L^−1^, 0.01 M PBS pH 7.2) as model protein focusing on extraction, clean-up, and elution. All experiments were run using 50 mg Magn-Humic and 2 mL samples spiked with 2 mg L^−1^ of each compound (CORT, E2, TST, and PROG) and, besides recovery, residual protein in the washing and in the eluate was monitored by the conventional Bradford assay (see Supplementary Material).

Concerning analytes adsorption on Magn-Humic, rotating plate shaker proved to favor extraction more consistently than vortex (data not shown), and not significantly different results were observed in going from 3 to 30 min contact; thus, 3 min was selected as the extraction time. In the washing step, 30% MeOH [21] caused a significant release of sorbed analytes, especially CORT, the steroid with the lowest partition coefficient (Log*P*) among the four probes (see Supplementary Material). Analytes recovery increased by reducing both the volume of the washing solution (from 2 to 1 mL) and the % of MeOH (from 30 to 5%, *v*/*v*). Considering that the residual protein in the eluate did not vary significantly, just the acidic washing (2 mL 2% *v*/*v* FA) was performed, affording removal of about 800 µg (40%) of adsorbed protein, with no loss of analytes.

Elution by 1 mL MeOH-ACN (1:1) allowed for the collection of a consistent fraction of steroids (65–80%) and a second elution, performed using the same eluent or 1 mL of MeOH, evidenced that the latter provided good elution and lower release of protein from the sorbent compared to the binary mixture. In the final eluate, obtained combining the two fractions, the residual protein was around 65 µg (against ca. 105 µg of the double elution with the mixture), corresponding to 0.3% of the BSA in the sample submitted to MSPE, as a result of the high protein exclusion (ca. 90%) joined to the acidic washing. At the same time, under these conditions, recovery was in the range 85–101% for all compounds. These findings account for a sorption process wherein the interaction with the sorbent displaces steroid–protein association [28], and elution by organic solvents induces the release of the potential fraction of protein-associated analytes [28].

To assess batch-to-batch reproducibility, additional recovery tests were done over non-consecutive days employing three independently synthesized batches of Magn-Humic. The observed RSD < 12% for the analytes recoveries is proof of reproducibility.

### 2.4. Optimization and Evaluation of MSPE in Biological Matrices

With the aim of maximizing recovery and method sensitivity in a real biological matrix, a DoE was planned to specifically focus on the performance of Magn-Humic in relation to the sample amount. Two factors were accordingly studied, namely sample volume (*x*_1_) and sorbent amount (*x*_2_) working on 1:4 diluted FBS samples (10 g L^−1^ proteins), spiked with 200 ng mL^−1^ of each analyte, in line with the experimental domain included in Supplementary Material. The mean multiclass recoveries observed under the different conditions are presented in Table 3, together with the residual protein in the MSPE eluate.

Recovery values were used as the experimental response (*y*) relative to each variable (*x*_i_), and they were modeled by the CAT software (Chemometric Agile Tool, available freely on the site of the Italian Group of Chemometrics) [29]) according to the following equation:

*y* = *b*_0_ + *b*_1_*x*_1_ + *b*_2_*x*_2_ + *b*_12_*x*_1_*x*_2_ (1)

**Figure 2:**
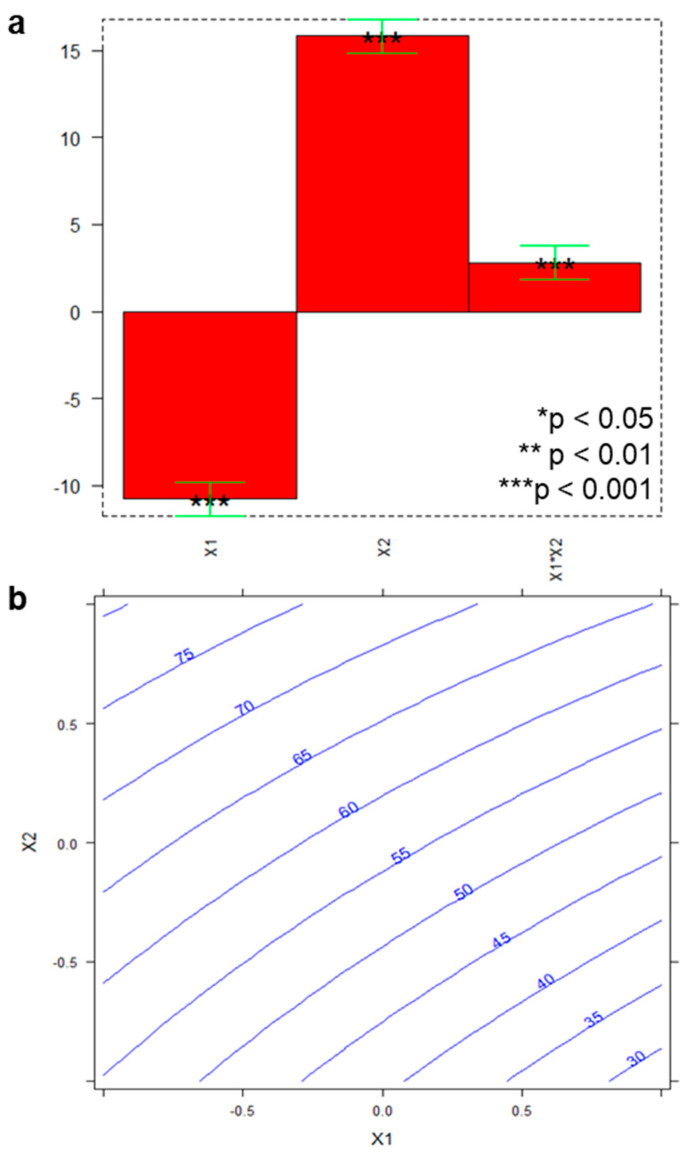
Design of experiments (DoE) results: (**a**) plot of the coefficients of the model (stars indicate the significance level of the coefficients, error bars indicate the confidence interval of the mean values for the coefficients, computed for *α* = 0.05); (**b**) response surfaces of predicted recovery as function of *x*_1_ (FBS volume) and *x*_2_ (sorbent amount).

The plot of the coefficients (*b*_i_) of the model and the response surface are gathered in Figure 2 (part a, and part b, respectively).

As shown in Figure 2a, both *x*_1_ and *x*_2_ proved to significantly affect recovery (*** *p* < 0.001); in particular, recovery was favored working with the lowest volume of biological matrix (*x*_1_) and the highest amount of magnetic material (*x*_2_). As well, interaction between the two factors (*x*_1_*x*_2_) resulted statistically relevant (*** *p* < 0.001), and, in line with the surface response graph (Figure 2b), recovery from about 80% upwards can be reached keeping *x*_1_ and *x*_2_ at the lowest and the highest level, respectively.

The model elaborated on the results from the MSPE tests, which yielded *y* = 57 − 11*x*_1_ + 16*x*_2_ + 3*x*_1_*x*_2_, was validated by the experiment at the test point *x*_1_ = 0 and *x*_2_ = 0, which means working with 750 µL FBS and 30 mg sorbent; indeed, the experimental recovery (65%) well matched the theoretical one predicted by the model (relative error 12%).

Under optimal conditions (50 mg Magn-Humic and 1 mL sample containing 250 µL FBS), which were also convenient in terms of clean-up (Table 3), recovery at 100 ng mL^−1^ was quantitative for all analytes, as shown in Table 4. To improve method sensitivity, additional recovery tests were done using a smaller volume of eluent, i.e., 2 × 0.5 mL, observing unchanged recovery.

To better cover the steroids concentration range typical of human plasma, further MSPE trials were undertaken in FBS to verify accuracy also at lower concentrations, ranging from 1 to 25 ng mL^−1^, and representative chromatograms are shown in Supplementary Material. As shown in Table 4, all compounds were quantified also at the lowest spike (1 ng mL^−1^, except E2, the steroid with the lowest instrumental sensitivity), in this case by evaporating to dryness the eluate and reconstituting the residue in 0.25 mL MeOH.

In this way, enrichment factor (EF) 4 was achieved with a substantial sensitivity gain compared with our earlier report [21] and a within-laboratory inter-day precision RSD < 14% (*n* = 3). As can be seen, quantitative recovery was gained for all compounds and only slightly lower (65%) for PREDLO just at the lowest spiking level.

The MSPE was then moved on 1:4 diluted plasma (~20 g L^−1^ proteins) spiked with 25 ng mL^−1^ with unchanged recovery, highlighting that the procedure works well also in biological fluids with a protein content higher than that of FBS, thus representing a simplified and effective alternative to the intensive sample treatment workflow required in bioanalysis [4].

### 2.5. Analytical Evaluation of the Method and Application to Bioanalysis

Selectivity is guaranteed by LC-MS with multiple-reaction monitoring (MRM) detection, which allows identification/quantification of the target compounds by using the two most intense transitions of each compound. Actually, no peaks of isobaric compounds or interfering species potentially co-extracted by Magn-Humic were evidenced at the steroids retention times in the chromatograms of FBS (blank matrix) MSPE eluate (Figure S5b).

The matrix-matched calibration, for quantitation of the concentrations expected after MSPE, was performed in the range 1–100 ng mL^−1^ (5–100 ng mL^−1^ for E2) by three independent calibration curves in the MSPE eluate from blank FBS and provided good linearity (r^2^ 0.9938–0.9999). Matrix effect (ME) resulted in an average signal suppression between 27 and 58% compared to the responses observed in pure solvent (MeOH), and it was quite well compensated by standard additions to the MSPE eluate. With regard to sensitivity, method detection and quantification limits (MDLs and MQLs) were in the range 0.02–0.3 ng mL^−1^ (0.8 ng mL^−1^ for E2) and 0.07–1 ng mL^−1^ (2.5 ng mL^−1^ for E2), respectively.

Trueness was assessed both in FBS and in human plasma at the ng mL^−1^ levels, obtaining satisfactory recoveries and good within-laboratory inter-day precision (Table 4) in agreement with criteria for analytical methods development at ng mL^−1^ levels [30,31].

No instrumental carry over was observed in the chromatograms of pure MeOH injected after each MSPE eluate, hence excluding cross-contamination. Carry over did not occur also in the MSPE step when recycling the sorbent, and reusability tests proved that Magn-Humic preserves its extraction performance for eight consecutive MSPE (recovery 65–107% at the eighth extraction).

**Figure 3:**
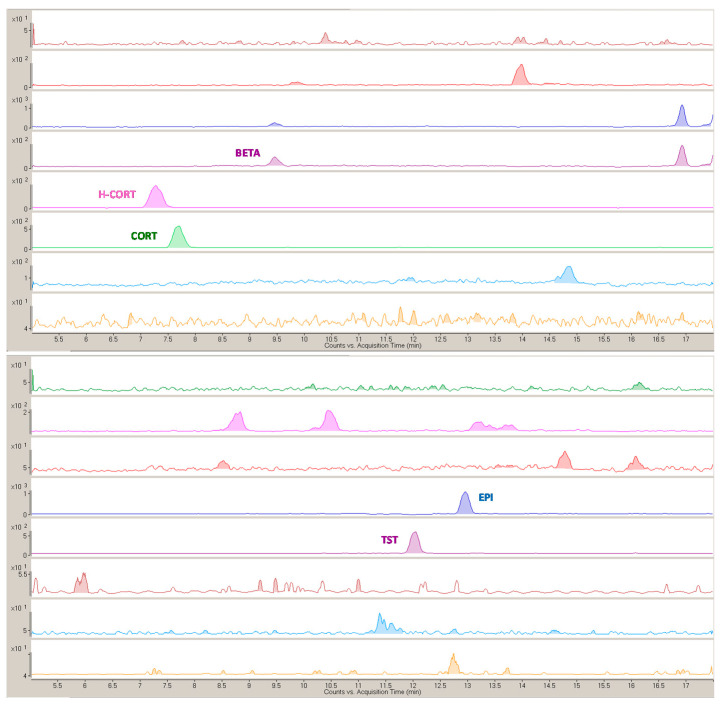
Representative multiple-reaction monitoring (MRM) chromatograms of the MSPE eluate from human plasma sample.

The method was applied to the analysis of three clinical human blind samples, and representative HPLC–MS/MS chromatographic profiles are reported in Figure 3.

Five steroids were quantified at concentrations from few to tens nanograms per milliliter, i.e., CORT (7–8 ng mL^−1^), H-CORT (34–71 ng mL^−1^), BETA (2 ng mL^−1^), EPI, and TST (1–2 ng mL^−1^), with RSD < 10% (*n* = 3). These plasmatic levels fall within the typical intervals reported in human plasma for such compounds [32,33,34]. The synthetic glucocorticoid BETA was found just in one of the three samples, and its presence is usually correlated to recent drug intake [32], while CORT and H-CORT were determined in all samples. The TST/EPI plasmatic concentrations ratio, strictly related to the urinary concentrations monitored in antidoping controls, resulted around 1 in the samples analyzed here.

### 2.6. Comparison with Literature and Critical Discussion

The sorbent here proposed, Magn-Humic, is attractive compared to the new materials recently used for (M)SPE ([6,11,33], Table 5), and the final extraction procedure, coupled to chromatographic separation, is a valid tool for multiclass steroids determination in human plasma.

Magn-Humic MSPE is easily done by a common-laboratory equipment and provides simultaneous extraction, clean-up, and pre-concentration in complex biological matrices avoiding protein precipitation [5,6,13], which is a cause of analyte loss [35] or large sample dilution [5,6]. Although the explorative 3D-printed sorbents newly proposed [11,33] can offer enhanced sample throughput in the 96-well plate format [11], the extraction here obtained is quantitative and definitely quicker. Compared to our earlier report on HA-C@silica [21], quantitation is here gained in plasma after 1:4 dilution (instead of 1:8); moreover, dilution is fully compensated by the EF. For its sensitivity, the method is suitable for therapeutic drug monitoring and pharmacokinetic studies.

The sorbent is a micron-sized composite suitable for MSPE thus avoiding use of packed cartridges, often affected by bed blockage or reduced flow rate [36], and vacuum systems/peristaltic pumps necessary for traditional column SPE. Meanwhile, the phase separation with an external magnetic field is rapid compared to centrifugation and filtration required for dispersive SPE using non-magnetic sorbents [36]. The sample preparation is carried out with just 50 mg sorbent and thus can be defined as micro-MSPE [37,38], and, at the same time, it requires smaller amounts of plasma (250 µL) than those (1–4 mL) generally used for MSPE of drugs in biological matrices [2]. An additional advantage is the reusability of the sorbent material; thus, 50 mg can be conveniently used for extraction of eight plasma samples.

## 3. Materials and Methods

### 3.1. Chemicals and Materials

Fe_3_O_4_ (50–100 nm, 20–50 m^2^ g^−1^), triethanolamine (TEOA, >99%), CTAB (≥99%), ethanol (96% *v*/*v*), HAs sodium salt (technical grade), BSA (> 98%), Bradford reagent (for micro and standard assays, 1–10 mg L^−1^ and 50–1400 mg L^−1^ proteins, respectively), nylon filters (0.2 µm), charcoal-stripped FBS, and high purity steroids standards were purchased from Sigma-Aldrich (Milan, Italy). Analytical grade H-PROG was supplied by Steroids (Cologno Monzese, Italy), and TRIAM and FLUO by Farmabios (Gropello Cairoli, Italy). Molecular structures and Log*P* values are shown in Supplementary Material. Technical grade acetone, HPLC gradient grade MeOH, ACN, and ultrapure water were provided by VWR (Milan, Italy). Tetraethyl orthosilicate (TEOS, 98%), FA (99%), NH_4_F (≥ 98%), Na_2_HPO_4_ (99%), and NaH_2_PO_4_·H_2_O (99%) were acquired from Carlo Erba Reagents (Milan, Italy).

MeOH steroids stock solutions (1000 µg mL^−1^) were stored in the dark (4 °C). Working solutions ≤ 1 µg mL^−1^ were prepared weekly in MeOH by dilution from a 10 µg mL^−1^ solution.

### 3.2. Preparation and Characterization of HA-C@SiO_2_@Fe_3_O_4_ (Magn-Humic)

Based on the results of our previous works [18,19,20,21] and the great advantages offered by MSPE [1,2], the idea of this study was to prepare a magnetic sorbent to be employed in a simplified extraction procedure in batch, i.e., dispersive MSPE. Considering two recent papers on magnetic porous silica prepared via sol-gel [23,24], this route was chosen to obtain an intermediate material (SiO_2_@Fe_3_O_4_) as the support for HAs (Appendix A) to prepare the magnetic sorbent HA-C@SiO_2_@Fe_3_O_4_, named Magn-Humic in the paper. In detail, 200 mg HAs were dissolved in 100 mL distilled water in a round-bottom flask, and 2 g SiO_2_@Fe_3_O_4_ or c-SiO_2_@Fe_3_O_4_ (air-calcined after sol-gel) were added, and the suspension was stirred for 2 min. Water was removed by rotary evaporator, and the obtained solid was pyrolyzed in an alumina combustion boat inside a quartz tube (600 °C, 1 h, N_2_ flow, heating 10 °C min^−1^, cooling 10 °C min^−1^) to convert HAs into a hydrophilic–lipophilic balanced carbonaceous phase [18,19]. Before use, Magn-Humic and c-Magn-Humic were washed in a filtering flask with plenty of distilled water until neutrality of the eluate. The batch-to-batch reproducibility was checked by recovery tests on three independent Magn-Humic preparations.

Microstructural characterizations were performed by a high-resolution scanning electron microscope (TESCAN Mira 3, Brno, Czech Republic), operating at 20.0 kV. Images were acquired on the powders after carbon (for SiO_2_@Fe_3_O_4_ and c-SiO_2_@Fe_3_O_4_) or platinum (for Magn-Humic and c-Magn-Humic) coatings, which were performed by either a Cressington 208C carbon coater or a Cressington HR 208, respectively (Watford, England, UK). The same instrumentation was used for compositional EDS analysis. TEM images were acquired by a JEOL JEM-1200EXIII instrument provided with a Mega View III CCD camera. Few milligrams samples were dispersed by sonication in about 3 mL water (Fe_3_O_4_), MeOH (SiO_2_@Fe_3_O_4_), or acetone (Magn-Humic), and then 10 µL of each suspension were deposited on grids and left to dry (room temperature).

TGA was performed using a Q5000 Instrument (TA Instruments Inc., New Castle, DE, USA). Each sample (10 mg) was heated (20 °C min^−1^) into a Pt pan from 25 up to 900 °C, using 100 mL min^−1^ air flow.

Surface areas were measured by the BET single point method using a Flowsorb II 2300 (Micromeritics, Norcross, GA, USA) apparatus. The sample was weighed and degassed at 80 °C (1.5 h) under a continuous stream of a N_2_-He (30:70) mixture, and then it was put in liquid N_2_ for gas adsorption.

### 3.3. Biological Samples

Being certified hormone-free FBS the recommended surrogate matrix [8,15,21], it was used as the blank for recovery tests at concentrations in the range 1–100 ng mL^−1^. Human plasma blind samples were provided by IRCCS Policlinico San Matteo (Pavia, Italy). Aliquots of the samples were frozen and stored at –20 °C. Before extraction, sub-samples were left to thaw at room temperature and vortexed for 20 s at 1400 rpm. In the case of recovery tests, samples were spiked, and after 30 min equilibration at room temperature, re-vortexed before MSPE.

### 3.4. MSPE Procedure for Simultaneous Extraction, Clean-Up, and Pre-concentration of Multiclass Steroids in Human Plasma

The MSPE procedure was carried out using 50 mg Magn-Humic in a self-standing 2 mL screw-cap glass vial. The material was conditioned using 2 mL phosphate buffer solution (PBS, 0.01 M, pH 7.2) by vortex (1400 rpm, 3 min), and then fast sedimentation of the solid was achieved by a neodymium magnet (Ø 10 mm, h 4 mm) placed under the vial. The liquid was withdrawn by a pipette, and the sample (1 mL from 1:4 plasma dilution, in PBS [11,21,25]) was added in the vial. Extraction was done on a rotating plate shaker (170 rpm, 3 min), the liquid was removed as above described, and washing was performed with 2 mL 2% (*v*/*v*) FA aqueous solution (vortex, 1400 rpm, 3 min). Analytes were eluted by 0.5 mL MeOH-ACN (1:1, *v*/*v*) and 0.5 mL MeOH, sequentially (vortex, 1400 rpm, 3 min). The two eluates were merged, filtered (0.2 µm), and evaporated to dryness under gentle N_2_ flow. The residue was re-dissolved in 0.25 mL MeOH for the HPLC–MS/MS analysis (see Appendix A). The overall time required for the extraction procedure is approximately 15 min. After use, the sorbent was contacted with 2 mL eluting solutions to avoid potential carryover, washed with 2 mL 2% (*v*/*v*) FA aqueous solution, and conditioned with 2 mL PBS for reusability tests.

### 3.5. MSPE Followed by HPLC–ESI-MS/MS: Analytical Parameters

Method selectivity was checked by analysis of blank samples (FBS) processed by all the steps of the analytical procedure described in the section above.

Linearity was assessed by ordinary linear least squares regression (OLLSR) on five-point calibration curves (1–100 ng mL^−1^) generated in both neat solvent, i.e., MeOH, and in FBS MSPE eluate after evaporation to dryness and reconstitution in 0.25 mL MeOH.

Matrix-matched calibration in the MSPE eluate was selected for quantification [18,20,21], at the same time compensating ME. This was calculated as:

(2) $$ME\;\left(\%\right)\;=\;\frac{b_{m}}{b_{s}}\;\times \;100$$

where *b_m_* and *b_s_* are the slopes of the matrix-matched calibration curve and the calibration line obtained in pure solvent, respectively [18,20,21]. MDLs and MQLs were calculated from the matrix-matched calibration curves, obtained in FBS MSPE extracts after N_2_ evaporation, as 3 and 10 times, respectively, the ratio between the baseline noise away from the peak tail and the regression line slope [18,20,21], considering that the pre-concentration (EF 4) compensated the initial sample dilution (1:4).

Accuracy was assessed in terms of trueness and precision. Due to the unavailability of certified reference materials (CRMs), trueness was verified by recovery tests (1–100 ng mL^−1^) in spiked FBS and human plasma samples by independent MSPE trials (*n* = 3), and the within-laboratory inter-day precision was evaluated based on RSD%. Instrumental carry-over was monitored by injections of MeOH, as control blank, after each chromatographic run.

## 4. Conclusions

The novel carbon-based magnetic material Magn-Humic has been prepared, characterized by various techniques, and successfully applied as sorbent for micro-MSPE of steroids in serum/plasma samples. Coupling the high sample protein exclusion and quantitative extraction afforded by using Magn-Humic to LC-MS^2^ analysis, satisfactory clean-up and multianalyte determination were possible with high selectivity. The sample treatment procedure, optimized by DoE, allows one to avoid large sample dilution and protein precipitation, requires small amount of sample, is simple, quick (around 15 min) and effective for multiclass determination of steroid hormones. The sorbent is reusable for repeated extractions, and it could be extended to environmental and food matrices.

## Figures and Tables

**Table 1:** Surface area values determined by Brunauer, Emmett, and Teller (BET) method (relative standard deviation (RSD) < 5%, *n* = 3).

Material	Surface Area (m^2^ g^−1^)
c-SiO_2_@Fe_3_O_4_	305
c-Magn-Humic	169
SiO_2_@Fe_3_O_4_	81
Magn-Humic	183

**Table 2:** Protein exclusion (%) observed in bovine serum albumin (BSA) solution (RSD < 6 %, *n* = 3).

Sorbent	% BSA Exclusion ^1^	Ref.
Magn-Humic	90(5)	This work
c-Magn-Humic	95(3)	This work
RACNTs	90(3)	[21]
HA-C@silica	86(2)	[21]

^1^ in parentheses the standard deviation.

**Table 3:** Mean multiclass recovery (*n* = 3), average of all analytes recoveries of each experiment, and residual protein in the magnetic solid-phase extraction (MSPE) eluate obtained in the conditions of the experimental plan.

Exp.	FBS Volume (µL), *x*_1_	Magn-Humic Amount (mg), *x*_2_	Recovery (%)	Residual Proteins (µg)
1	250	10	55	57
2	1250	10	28	123
3	250	50	81	133
4	1250	50	65	237

**Table 4:** Recoveries obtained in FBS by the optimized MSPE procedure followed by high-performance liquid chromatography coupled with mass spectrometry (HPLC–MS/MS) (*n* = 3).

		Mean Recovery (%) ^1^			
	Spike (ng mL^−1^)	100	25	5	1 ^2^
PREDLO		95	84	87	65
PRED		109	98	97	107
H-CORT		80	80	71	70
CORT		91	87	97	70
BETA		97	94	104	122
DEXA		97	96	80	75
TRIAM		100	95	110	104
E2		106	115	109 ^2^	n.q. ^3^
TST		84	81	95	92
EPI		84	82	94	94
EE2		89	82	88	97
E1		86	89	105	90
H-PROG		105	90	84	96
FLUO		75	91	97	106
PROG		88	89	80	84
M-PROG		98	101	99	82

^1^ RSDs < 14%, *n* = 3. ^2^ evaporation of the MSPE eluate and reconstitution in 0.25 mL MeOH (EF 4) before analysis. ^3^ n.q., not quantifiable at this concentration level.

**Table 5:** Comparison with current analytical methods involving (M)SPE followed by chromatographic separation for steroids multiclass determination in human plasma.

Steroids, Analysed Number and Classes	Plasma Volume (µL)	Protein Precipitation	Centrifugation	Dilution	Extraction Technique	Sorbent (amount, mg)	Elution	Derivatization	Recovery (%)	RSD (%)	MQLs (ng mL^−1^)	Analysis	Ref.
10: 4 estrogens, 1 androgen, 3 progestagens, 2 glucocorticoids	2000	MeOH	n.a. ^1^	H_2_O (+36 mL)	SPE	C18 (500 mg)	2 mL MeOH	-	85.3–99.9	0.2–8.3	4–157 (MDLs)	HPLC–UV	[5]
19: 3 estrogens, 6 androgens, 4 progestagens, 6 glucocorticoids	400	-	-	H_2_O (+4 mL)	SPE	C18 (500 mg)	5 mL MeOH- H_2_O (80:20)	-	93.9–137.3	1.5–15.6	0.055–0.530	HPLC–MS	[8]
7: 2 estrogens, 4 androgens, 1 progestagen	495	0.1 % FA	20,220× *g*, 10 min, 4 °C	H_2_O (to 2 mL)	SPE	C18 (500 mg)	3 mL ethylacetate	Step 1. 30 min, 30 °C Step 2. 30 min, 40 °C	69.2–100	1.6–35.5	0.01–5	GC-MS	[13]
16: 3 estrogens, 2 androgens, 3 progestagens, 8 glucocorticoids	250	-	-	PBS (to 2 mL)	SPE	HA-C@silica ^2^ (100 mg)	1 mL MeOH-ACN (1:1)	-	64–118	< 15	2–10 (15 for E2)	HPLC–MS	[21]
7: 1 estrogen, 3 androgens, 2 glucocorticoids, 1 mineralcorticoid	n.a.	-	-	n.a.	dispersive SPE	3D-printed LayFOMM 60^®^	ACN-H_2_O (80:20), 75 min, 750 rpm	-	19.3–84.9	1.44–9.46	3-10	HPLC–MS	[33]
5: 1 estrogen, 2 androgens, 1 glucocorticoid, 1 mineralcorticoid	300	-	-	PBS (to 1.5 mL)	96-well plate SPE	3D-printed LayFOMM 60^®^	ACN-H_2_O (80:20), 75 min	-	2.05–38.07	3.02–18.14	n.a.	HPLC–MS	[11]
2: 1 androgen, 1 progestagen	n.a.	ACN	3000 rpm, 30 min	H_2_O (to 50 mL)	MSPE	TMSPT-MNP@Au ^3^ (50 mg)	1 mL MeOH, 3 min	-	94.5–99.1	3.49–4.19	0.05–0.07 (MDLs)	HPLC–UV	[6]
16: 3 estrogens, 2 androgens, 3 progestagens, 8 glucocorticoids	250	-	-	PBS (to 1 mL)	MSPE	Magn-Humic (50 mg)	0.5 mL MeOH-ACN (1:1) + 0.5 mL MeOH (vortex, 1400 rpm, 3 min)	-	65–122	5–14	0.07–1 (2.5 for E2)	HPLC–MS	This work

^1^ n.a., not available. ^2^ HA-C@silica, silica-supported carbon from humic acids pyrolysis. ^3^ TMSPT-MNP@Au, Au nanoparticles grafted on 3-(trimethoxysilyl)-1-propanethiol modified Fe_3_O_4_ magnetic nanoparticles.

## Data Availability

The data presented in this study are available on request from the corresponding author.

## Supplementary Materials

The following are available online, Figure S1: Representative TEM images acquired on (a) pristine Fe_3_O_4_ (50 kx) and (b1, b2) SiO_2_@Fe_3_O_4_ (150 kx); Figure S2: Representative SEM images acquired on (a) Magn-Humic and (b) c-Magn-Humic; Figure S3: TGA profiles recorded on (a) c-SiO_2_@Fe_3_O_4_, (b) c-Magn-Humic, (c) SiO_2_@Fe_3_O_4_, and (d) SiO_2_@Fe_3_O_4_ after isothermal pretreatment for 12 h at 320 °C, (e) Magn-Humic; Figure S4: Mean calibration curve (*n* = 6) for the Bradford assay; Figure S5: MRM chromatograms of MSPE eluates from (a) FBS spiked with 5 ng mL^−1^ of each compound before extraction and (b) unspiked FBS; Table S1: Molecular structures and Log*P* values of the studied steroids (data from https://hmdb.ca/, accessed on 26 February 2021); Table S2: Compositional results collected on the materials obtained after sol-gel compared to pristine magnetite; Table S3: MRM conditions for HPLC–ESI-MS/MS analysis of the steroids; Table S4: Experimental domain of the variables selected for the 2^2^ factorial design.

## Sample Availability

Samples of the compounds are not available from the authors.

## Appendix A

### A.1. Preparation of SiO_2_@Fe_3_O_4_

1 g Fe_3_O_4_ was suspended in 90 mL water and sonicated for 30 min. Subsequently, 1.7 g of CTAB and 1 g of TEOA were added, and the mixture was continuously mixed by an overhead mechanical stirrer for 1 h at 80 °C using a thermostatically controlled bath (in a fumehood). Then, 14 mL TEOS were rapidly added, and the reaction mixture was maintained under stirring at 80 °C for 2 h. The obtained SiO_2_@Fe_3_O_4_ was recovered by filtration, washed with 50 mL ethanol, dried in oven (60 °C, 24 h), and used in the subsequent step, eventually after calcination (c-SiO_2_@Fe_3_O_4_) at 540 °C, 7 h [23].

### A.2 HPLC–UV

The chromatographic apparatus consisted of a Shimadzu (Milan, Italy) LC-20AT solvent delivery module equipped with a DGU-20A3 degasser and interfaced with an SPD-20A detector. A Scharlab Kroma Phase 100 C18 (250 × 4.6 mm, 5 μm) column coupled with a Supelco Supelguard Ascentis C18 (20 × 2.1 mm, 5 μm) guard-column was used. After an equilibration period of 3 min, 20 μL of each sample was manually injected in the system. The mobile phase was (A) water and (B) ACN, flow rate of 1 mL min^−1^. Elution program: linear gradient from 30 to 90% B until 12 min, then to 95% B until 15 min, finally to 100% B until 18 min (kept for 5 min). The detection wavelengths were 225 nm for estrogens and 242 nm for the other compounds. Calibration standard solutions (1–9 mg L^−1^) were prepared in MeOH-ACN (1:1, *v*/*v*) obtaining good linearity (r^2^ > 0.9923).

### A.3 HPLC–ESI-MS/MS

The target substances were analyzed with a HPLC apparatus Agilent 1260 Infinity coupled with an Agilent 6460C MS spectrometer ESI-MS/MS system (Cernusco sul Naviglio, Italy). The MS operating parameters, optimized by Agilent Mass Hunter Source Optimizer Software (Agilent, USA), were the following: drying gas (N_2_) temperature 350 °C; drying gas flow 12 L min^−1^; nebulizer 50 psi; sheath gas temperature 400 °C; sheath gas flow 12 L min^−1^; capillary voltage 4000 V positive, 3000 *V* negative; nozzle voltage 0 *V* positive, 1500 *V* negative; electron multiplier voltage (EMV) 200 V positive, 0 *V* negative; and cell accelerated voltage 1 and 4 *V* for negative and positive mode, respectively. Quantitative analysis was performed in MRM mode, using the most intense transitions from precursor ion to product ions for each analyte (see Table S3).
