# Supplementary material for: Magnetic Micro-Solid-Phase Extraction Using a Novel Carbon-Based Composite Coupled with HPLC–MS/MS for Steroid Multiclass Determination in Human Plasma
Source: Molecules. 2021 Apr 3;26(7):2061. doi: 10.3390/molecules26072061 (PMC8038327; doi:10.3390/molecules26072061)
Supplement: Supplementary file 1 [file molecules-26-02061-s001.pdf]

Supplementary Information

Magnetic micro-solid-phase extraction using a novel carbon-based composite coupled with HPLC-MS/MS for steroid multiclass determination in human plasma

Andrea Speltini[1,*], Francesca Merlo[2], Federica Maraschi[2], Giorgio Marrubini[1], Anna Faravelli[2] and Antonella Profumo[2,*]

[1] Department of Drug Sciences, University of Pavia, via Taramelli 12, 27100 Pavia, Italy; andrea.speltini@unipv.it; giorgio.marrubini@unipv.it
[2] Department of Chemistry, University of Pavia, via Taramelli 12, 27100 Pavia, Italy; francesca.merlo02@universitadipavia.it; federica.maraschi@unipv.it; anna.faravelli01@universitadipavia.it; antonella.profumo@unipv.it
* Correspondence: andrea.speltini@unipv.it (A.S.); antonella.profumo@unipv.it (A.P.) Tel.: +39 0382 987349

Target analytes:

Table S1. Molecular structures and Log*P* values of the studied steroids (data from https://hmdb.ca/).

Steroid	Molecular structure	Log*P*
PREDLO	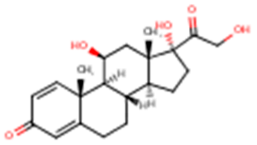	1.66
PRED	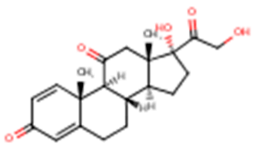	2.07
H-CORT	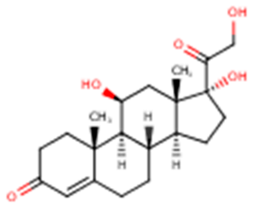	1.79

CORT	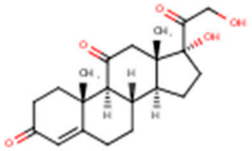	1.98
BETA	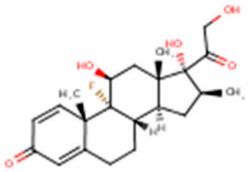	1.93
DEXA	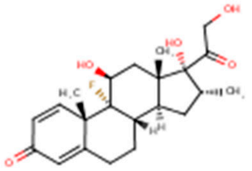	1.93
TRIAM	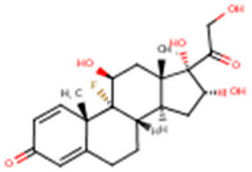	0.84
E2	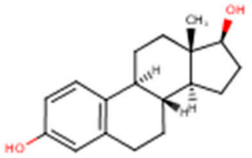	3.57
TST	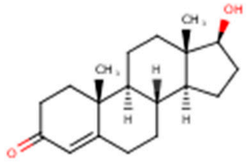	2.99
EPI	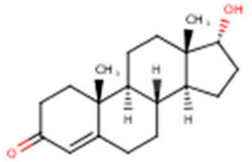	2.99

EE2	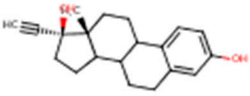	3.63
E1	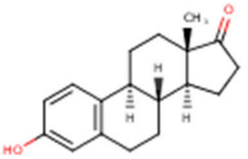	4.03
H-PROG	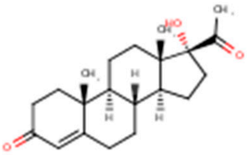	2.99
FLUO [a]	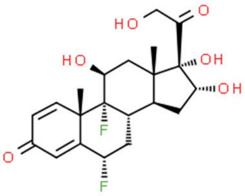	0.58
PROG	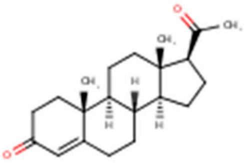	3.58
M-PROG	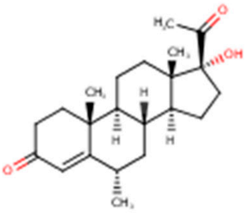	3.52

[a] data from https://www.chemspider.com/

TEM images:

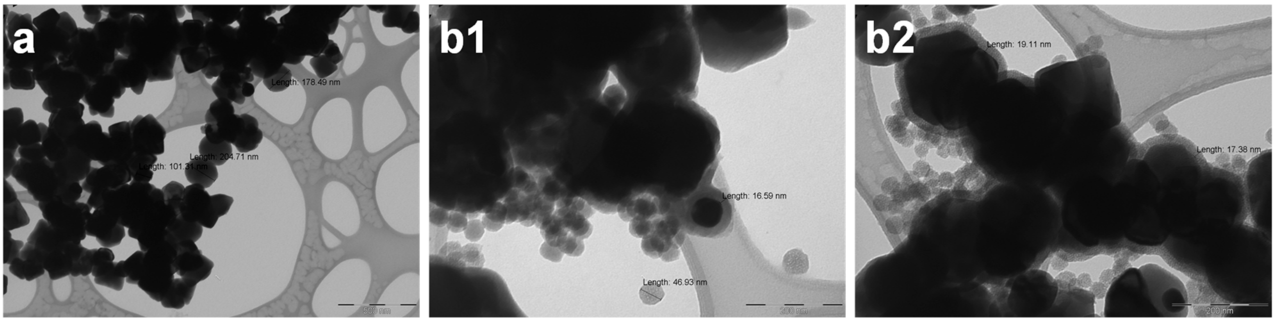

Figure S1. Representative TEM images acquired on (**a**) pristine Fe_3O_4 (50 kx) and (**b1**, **b2**) SiO_2@Fe_3O_4 (150 kx). The Fe_3O_4 particles (black) are coated by silica as both a continuous layer and nanometric spheres (grey); the thickness of the silica layer and the dimensions of the silica and magnetite particles are reported in the figure.

SEM images:

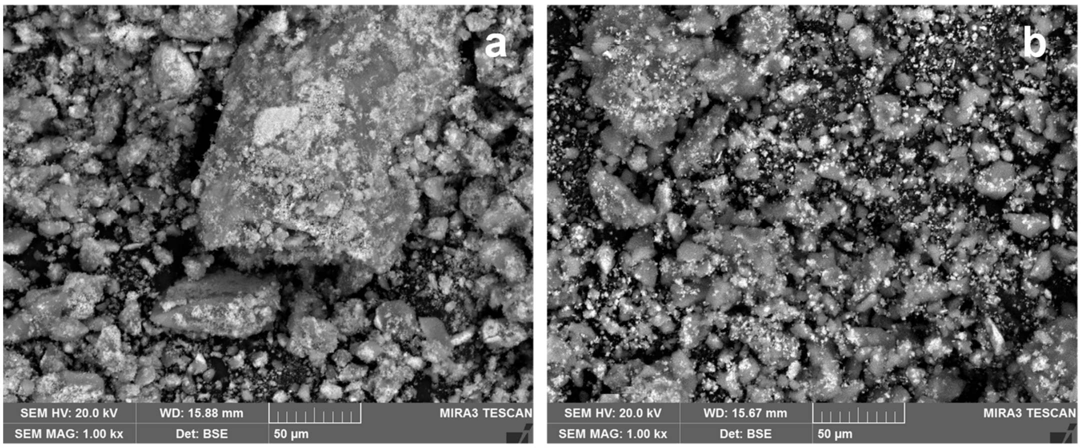

Figure S2. Representative SEM images acquired on (**a**) Magn-Humic and (**b**) c-Magn-Humic.

TGA profiles:

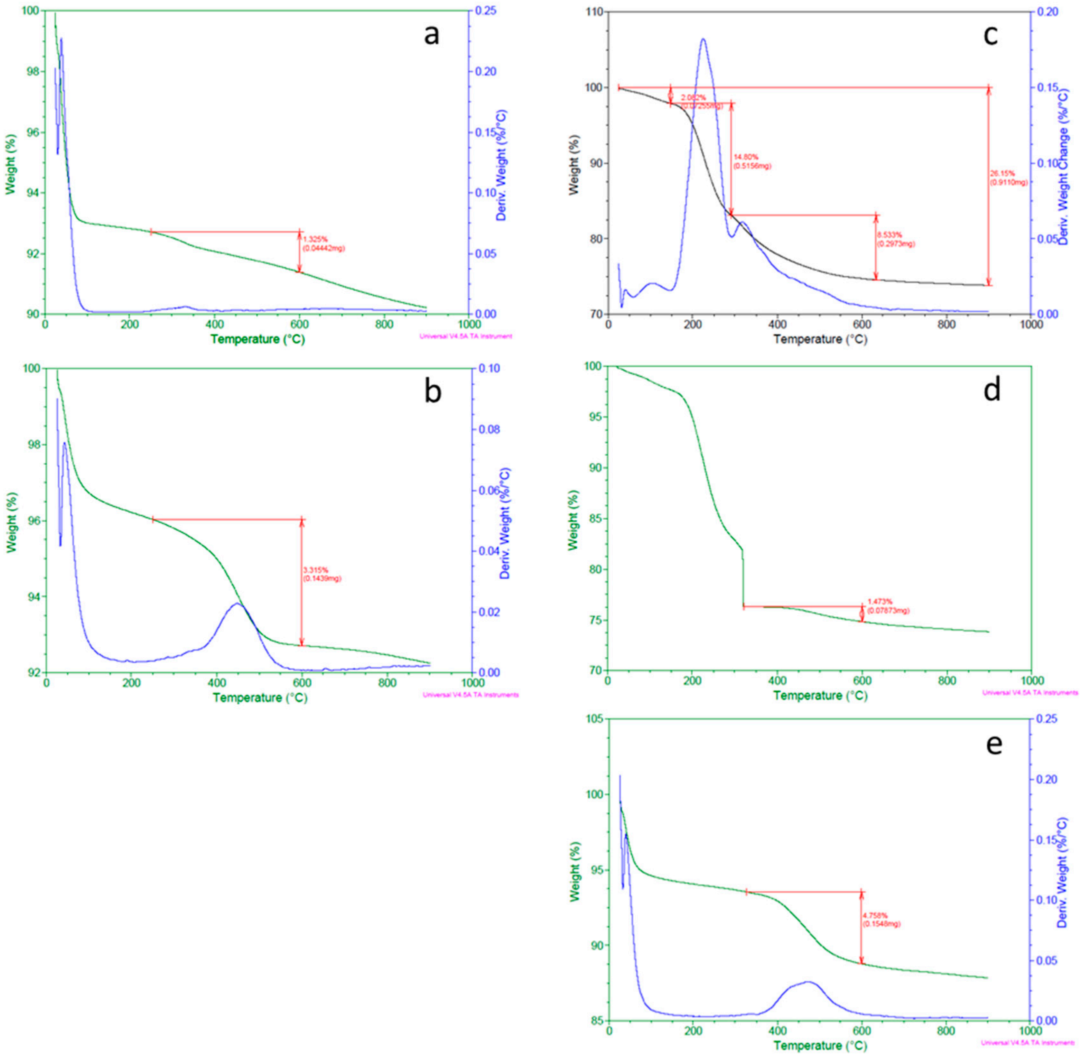

Figure S3. TGA profiles recorded on (**a**) c-SiO_2@Fe_3O_4, (**b**) c-Magn-Humic, (**c**) SiO_2@Fe_3O_4, (**d**) SiO_2@Fe_3O_4 after isothermal pre-treatment for 12 h at 320 °C, (**e**) Magn-Humic.

EDS analysis:

Table S2. Compositional results collected on the materials obtained after sol-gel compared to pristine magnetite.

**Material**	**Si/O**	**Si/Fe**
Fe_3O_4	0.00	0.00
SiO_2@Fe_3O_4	0.26	3.54
c-SiO_2@Fe_3O_4	0.20	3.96

MRM conditions

Table S3. MRM conditions for HPLC-ESI-MS/MS analysis of the steroids.

Compound	Precursor ion* (m/z)	Product ion (m/z)	Dwell time	Fragmentor energy (V)	Collision energy (V)	Polarity
PREDLO	343	147	15	112	20	Positive
		91	15	112	72	
H-CORT	363	121	15	124	20	Positive
		91	15	124	68	
PRED	359	341	15	76	4	Positive
		147	15	76	24	
CORT	361	163	15	130	20	Positive
		105	15	130	48	
BETA	373	355	15	82	8	Positive
		147	15	82	24	
DEXA	373	355	15	76	4	Positive
		91	15	76	80	
TRIAM	415	397	15	130	8	Positive
		91	15	130	80	
FLUO	495	475	15	88	0	Positive
		337	15	88	8	
E2	271	183	100	166	50	Negative
		143	100	166	64	
TST	289	109	15	76	24	Positive
		97	15	76	20	
EPI	289	253	15	100	20	Positive
		109	15	100	25	
		97	15	100	20	

EE2	295	145	50	154	44	Negative
		143	50	154	68	
E1	269	145	15	148	44	Negative
		143	15	148	60	
H-PROG	331	109	15	118	28	Positive
		97	15	118	24	
PROG	315	109	15	94	24	Positive
		97	15	94	20	
M-PROG	387	327	15	106	8	Positive
		123	15	106	24	

*$[M\text{-}H]^-$ adduct for negative ion and $[M+H]^+$ adduct for positive ion.

Analytes are detected in the same chromatographic run by setting a polarity-switching tool. The chromatographic separation was done on a Zorbax Eclipse Plus C18 column (4.6 × 100 mm, 3.5 µm), equipped with a similar guard-column (Robusta C18 4.6 × 10 mm, 5 µm), maintained at 30 °C. Gradient elution (0.5 mL min^{-1}) was performed by (A) aqueous 1 mM NH_4F and (B) ACN: 30 % B for 0.5 min, linear gradient to 85 % B in 11.5 min, isocratic 85 % B for 5 min, to 98 % B in 0.5 min, hold for 2 min (washing step); the initial conditions (30 % B) were returned by 7-min equilibration time. The injection volume was 10 µL.

Bradford assay:

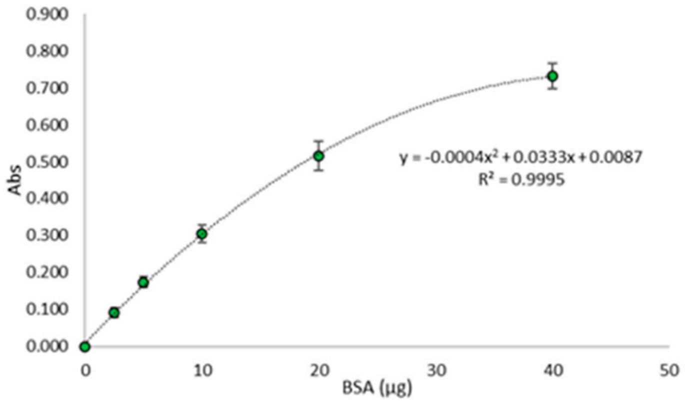

Figure S4. Mean calibration curve (*n*=6) for the Bradford assay (0-40 µg protein, starting from a 50 mg L^{-1} BSA standard solution in 0.01 M phosphate buffer, pH 7.2); spectrophotometric determination at λ_{max} 595 nm, 1 cm-optical path cuvette.

Design of experiments:

Table S4. Experimental domain of the variables selected for the 2^2 factorial design.

Variable	Levels	
	-1	+1
FBS volume (µL), x_1	2	5
Magn-Humic amount (mg), x_2	10	20

MRM chromatograms:

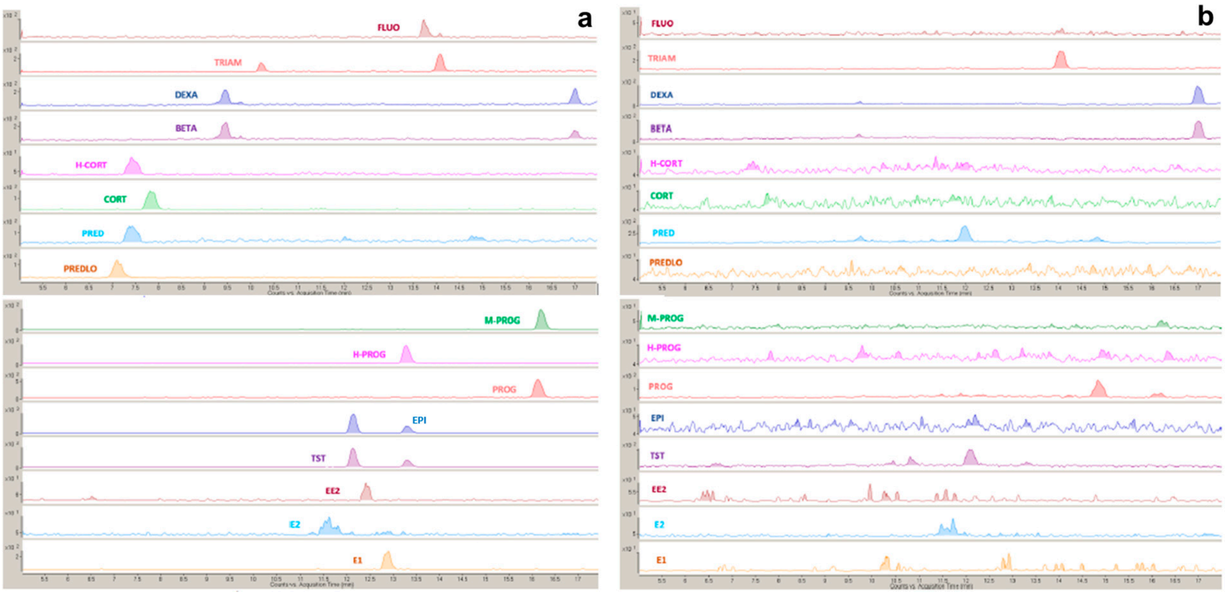

Figure S5. MRM chromatograms of MSPE eluates from (**a**) FBS spiked with 5 ng mL^{-1} of each compound before extraction and (**b**) unspiked FBS.
